# Epidemiology of 10-year paediatric renal biopsies in the region of southern Croatia

**DOI:** 10.1186/s12882-020-01727-7

**Published:** 2020-02-26

**Authors:** Adela Arapović, Katarina Vukojević, Natalija Filipović, Merica Glavina Durdov, Danica Ljubanović-Galešić, Mirna Saraga-Babić, Sandra Prgomet, Ana Simičić Majce, Anja Belavić, Dijana Borić Škaro, Dragan Ljutić, Marijan Saraga

**Affiliations:** 1 Department of Pediatrics, University Hospital Centre Split, 21000 Split, Croatia; 2 Department of Anatomy, Histology and Embryology, University of Split School of Medicine, Šoltanska 2, 21000 Split, Croatia; 3 Department of Pathology, University Hospital Centre Split, 21000 Split, Croatia; 4 University of Split School of Medicine, 21000 Split, Croatia; 5 Department of Pathology, Clinical Hospital Dubrava, 10000 Zagreb, Croatia; 6 University of Zagreb School of Medicine, 10000 Zagreb, Croatia; 7 Division for School Medicine, Mental Health and Addiction Prevention, Croatian Institue of Public Health, 10000 Zagreb, Croatia; 8 Department of Nephrology, University Hospital Centre Split, 21000 Split, Croatia

**Keywords:** Epidemiology, Registries, Renal biopsy, Nephrotic syndrome

## Abstract

**Background:**

Information about renal diseases in children is available from national registries of renal biopsies. Aim of the study was to compare the clinical presentation of glomerular diseases and tubulointerstitial space diseases with pathohistological diagnosis of indicated renal biopsies from pediatric population in the Croatian region of Dalmatia.

**Methods:**

Out of 231 pediatric patients with suspected glomerular and tubulointerstitial diseases**,** 54 underwent ultrasound-guided renal biopsy at University Hospital of Split. Kidney allograft biopsy, and re-biopsy were excluded. The biopsy sections were examined under light microscopy, immunofluorescence and electron microscopy. The data was reviewed to determine the pathohistological spectrum and clinicopathologic correlations**.** We retrospectively analyzed kidney biopsy data from 2008 to 2017 and compared them to that between 1995 and 2005.

**Results:**

The mean age of patients was 9.84 ± 5.4 years. Male:female ratio was 1.2:1. The main indications for biopsy were *pure nephrotic syndrome without hematuria* (25.9%), *non-nephrotic proteinuria with haematuria* (22.2%), *nephritic syndrome with nephrotic proteinuria* (18.5%), and *isolated hematuria* (16.7%). The most common pathohistological findings were IgA nephropathy (IgAN, 24.1%), minimal change disease (MCD, 16.7%), Henoch-Schönlein purpura glomerulonephritis (HSPN, 14.8%), Alport syndrome and focal segmental glomerulosclerosis (AS and FSGS, 11.1% each), tubulointerstitial nephritis and membranous glomerulopathy (TIN and MGN, 3.7% each), while other cases were diagnosed rarely.

**Conclusions:**

Changes in epidemiology of renal diseases in children between the analyzed periods showed an increasing trend of IgAN, MCD, HSPN, AS and FSGS, while mesangioproliferative glomerulonephritis (MesPGN) and endoproliferative glomerulonephritis (EDGN) showed a decreasing trend that can be explained with the new pathohistological classification.

## Background

Renal diseases are very important as they significantly contribute to morbidity of children. National registries of renal biopsies showed a variety of renal diseases and different epidemiology worldwide [1–6]. Renal biopsies are still very important for renal diseases management in children [7–22].

In our clinical experience we observed that the occurrence of certain glomerular and tubulointerstitial diseases changed in the last 10 years and also that the indication of renal biopsies in children had changed. Therefore, the aim of our study was to compare the clinical presentation of glomerular diseases and tubulointerstitial space diseases with pathohistological diagnosis of indicated renal biopsies in the pediatric population. Here we present a summary of clinico-pathological associations of kidney diseases worldwide, in order to relate renal pathohistological diagnoses (PHD) with our previous results regarding the period between 1995 and 2005 [9]. We also analyzed changes in epidemiological trends of kidney diseases and compared them with the data from other national registries of kidney biopsies in children. The differences between kidney diseases in Croatia and data from other studies could be useful in paediatrics for understanding the current state of children’s renal pathology in south-eastern European countries and a base for possible formation of a National Registry of Renal Biopsies in Children in Croatia.

## Methods

This retrospective study was conducted at University Hospital of Split (UHC Split) at the Department of Pediatrics in accordance with the Helsinki Declaration. Ethical Approval was granted from the Ethical Committee of UHC Split. All patients with glomerular and tubulointerstitial diseases admitted at the Department of Pediatric Nephrology between 2008 and 2017 were evaluated (231 patients) and indications for renal biopsies were determined by our Department’s protocols. Kidney biopsy was performed using an automated spring-loaded biopsy instrument under ultrasound guidance. All biopsy samples were analyzed by light and immunofluorescence microscopy. The polyclonal antisera were used against human IgG, IgA, IgM, C1q, C3 and C4. All biopsies samples were analysed by electron microscopy. Two experienced renal pathologists examined all renal biopsies independently according the WHO recommendations and Oxford classification in cases of IgA nephropathy (IgAN) [23, 24]. No transplant biopsies and re-biopsies were included in this study. There have been several patients with multiple biopsies with the same disease, but we included only data from the first one. Additionally, all repeated biopsies happened during the period of interest and they did not overlap in time with the previously reported cohorts from period 1995–2005. Patients who underwent renal biopsy were supervised for 48 h for possible complications. Various renal clinical manifestations were analysed in regard to pathohistological diagnosis (PHD).

Criteria for renal biopsy included: pure nephrotic syndrome without hematuria, non-nephrotic proteinuria with hematuria, nephritic syndrome with nephrotic proteinuria, isolated hematuria, acute renal failure, chronic renal failure, acute nephritic syndrome and non-nephrotic persistent proteinuria as we described previously [9]. Arterial hypertension in children was defined as average systolic or diastolic blood pressure greater than or equal to the 95th percentile for age, gender and height.

Steroid therapy response was classified as; steroid sensitive (SSNS), steroid dependent (SDNS) steroid resistant (SRNS), frequently relapsing (FRNS), infrequently relapsing (IFRNS), and complete remission (CRNS) [25–27]. The response to steroid therapy was classified as; steroid sensitive (SSNS) (disappearance of proteinuria within 8 weeks of oral prednisone therapy at a dose of 60 mg/m^2^/day), steroid dependent (SDNS) (tendency to relapse during prednisone therapy or within 2 weeks of discontinuation), steroid resistant (SRNS) (a failure for remission to 8 consecutive weeks of treatment with oral prednisone at 60 mg/m^2^/day followed by 3 pulse doses of methylprednisolone), frequently relapsing (FRNS) (2 or more relapses per 6 months of the initial response or 4 or more relapses per any 12-month), infrequently relapsing (IFRNS) (less than 2 relapses per 6 months or less than 4 relapses per any 12-month), remission (urinary protein excretion < 4 mg/m^2^/h) [25–27].

### Statistical analysis

Chi-square test was used to determine statistical differences between the analyzed periods from 1995 to 2005, and from 2008 to 2017 (GraphPad Software, La Jolla, CA, USA). Trends and breakpoints in time series were analysed by jointpoint regression using BIC as model selection criteria. Jointpoint regression was done in Joinpoint Regression Program (Version 4.7.0.0. February, 2019; Statistical Research and Applications Branch, National Cancer Institute, Calverton, MD, USA). If the trend and breakpoint analysis of time series suggested unsegmented linear model as the best, then a Poisson regression was done to estimate the model coefficients; Poisson regression was done in Past3 software. Statistical significance was *p* < 0.05.

## Results

**Fig. 1:**
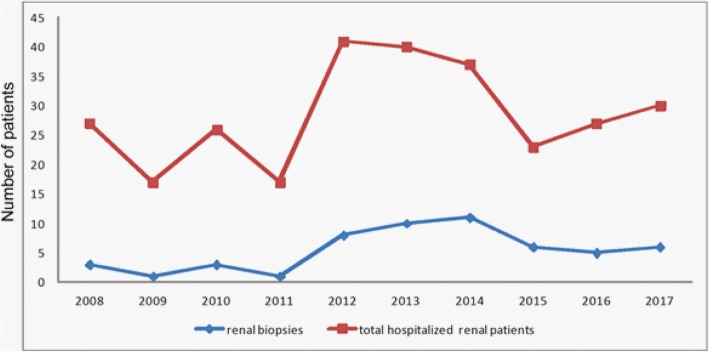
Number of total hospitalized renal patients which presented with glomerular or tubulointerstitial disease and renal biopsies for each year

Out of 231 patients with glomerular and tubulointerstitial diseases there were 54 (23.4%) patients (29 boys and 25 girls under 18 years of age) that had indication for renal biopsy (Fig. 1, Table 1). At the time of biopsy, the median age was 9.84 ± 5.4 years). The females had mean age 9.84 ± 5.4 years and males 9.96 ± 4.9 years.

**Table 1:** Basic patient characteristics

Patient	age (year)	serum creatinine (μmol/L)	eGFR (Schwartz) (mL/min/1.73m^2^)	protein excretion levels (g/day)	serum albumin (g/L)	edema	haematuria	hypertensio arterialis
1	2	33	98.47	3.1	13	yes	no	no
2	12	46	130.17	0.105	41	no	yes	no
3	1	504	5.94	1.6	24	yes	yes	yes
4	15	60	96.14	1.8	40	no	yes	yes
5	11	55	91.61	3.4	23	yes	no	yes
6	6	50	87.63	3.32	24	yes	yes	yes
7	2	31	105.41	1.74	23	yes	yes	no
8	15	58	117.08	4.41	23	yes	no	no
9	16	61	111.33	0.071	40	no	yes	no
10	13	52	120.76	0.311	38	no	yes	yes
11	18	374	17.47	12.2	24	yes	yes	yes
12	1	43	120.57	0.523	41	no	yes	no
13	15	51	132.08	2.5	42	no	yes	yes
14	17	127	45.42	0.143	36	no	no	yes
15	14	44	147.7	0.078	38	no	yes	no
16	5	42	99.1	1.75	24	yes	no	no
17	10	61	89.78	1.71	31	yes	yes	no
18	15	57	112.41	0.778	34	no	yes	yes
19	15	50	125.59	2.49	34	yes	yes	no
20	13	54	112.91	0.967	42	no	yes	no
21	3	28	129.09	13.9	13	yes	no	no
22	13	46	123.42	0.902	44	no	no	yes
23	3	767	4.47	2.64	35	no	no	yes
24	7	50	90.55	0.054	39	no	yes	no
25	16	62	88.63	52.9	11	yes	yes	no
26	4	39	103.91	0.056	36	no	yes	no
27	17	72	91.28	4.0	30	yes	no	no
28	9	43	121.42	6.21	29	yes	yes	yes
29	6	44	94.18	0.097	47	no	yes	no
30	15	70	91.80	0.06	47	no	yes	no
31	17	76	82.39	6.01	26	yes	yes	no
32	13	66	89.62	3.7	29	yes	yes	yes
33	9	44	116.12	0.270	35	no	yes	yes
34	4	35	108.9	12.26	16	yes	no	no
35	16	76	87.91	7.4	22	yes	yes	no
36	4	33	119.49	2.8	21	yes	no	no
37	3	35	94.93	0.246	39	no	yes	no
38	7	34	144.32	3.2	17	yes	no	no
39	12	61	89.78	0.174	42	no	yes	yes
40	6	34	120.81	0.780	22	yes	yes	no
41	13	90	62.47	3.28	31	yes	yes	no
42	14	62	94.81	3.5	35	no	no	no
43	12	45	140.36	4.0	33	yes	yes	no
44	10	60	91.93	0.985	37	no	yes	no
45	12	49	116.24	0.108	39	no	yes	no
46	17	68	91.28	0.564	38	no	yes	no
47	10	595	9.27	8.4	25	yes	yes	yes
48	6	39	105.78	0.086	33	no	yes	no
49	11	46	111.5	3.2	17	yes	no	no
50	6	46	94.85	2.5	24	yes	no	no
51	17	57	107.61	4.5	21	yes	no	no
52	4	390	10.29	1.2	31	yes	yes	yes
53	4	34	106.5	28.5	12	yes	yes	yes
54	6	123	38.58	0.9	38	no	no	no

### Indications for renal biopsy

*Pure nephrotic syndrome without hematuria* (NS) was the most common reason for renal biopsy that presented in 25.9% of cases. *Non-nephrotic proteinuria with hematuria* was observed in 22.2% of biopsied cases. *Nephritic syndrome with nephrotic proteinuria* was found in 18.5%, and *isolated hematuria* was found in 16.7%. *Acute renal failure* was found in 7.4%, while *chronic renal failure* was observed in 5.5% of biopsied cases. *Acute nephritic syndrome* and *non-nephrotic proteinuria* were relatively rare indications for renal biopsy (Table 2).

**Table 2:** Clinical indications for renal biopsy, and distribution of pathohistological diagnosis in all biopsied patients (*N* = 54)

Syndrome	Number	Percentage
Isolated haematuria	9	16.7
Non-nephrotic proteinuria	1	1.9
Non-nephrotic proteinuria with haematuria	12	22.2
Pure nephrotic syndrome without hematuria	14	25.9
Nephritic syndrome with nephrotic proteinuria	10	18.5
Acute renal failure	4	7.4
Chronic renal failure	3	5.5
Acute nephritic syndrome	1	1.9
Pathohistological diagnoses		
Mesangioproliferative glomerulonephritis (MesPGN)	1	1.9
IgA nephropathy (IgAN)	13	24.1
Henoch-Schӧnlein purpura glomerulonephritis (HSPN)	8	14.8
Tubulointerstitial nephritis (TIN)	2	3.7
Focal segmental glomerulosclerosis (FSGS)	6	11.1
Endoproliferative glomerulonephritis (EDGN)	1	1.9
Alport syndrome (AS)	6	11.1
Thin basement membrane nephropathy (TBMN)	1	1.9
Minimal change disease (MCD)	9	16.7
Crescentic GN	1	1.9
Membranous glomerulopathy (MGN)	2	3.7
Lupus nephritis (LN)	1	1.9
C1q nephropathy (C1qN)	1	1.9
Focal segmental necrotizing glomerulonephritis (FSNGN)	1	1.9
C3 glomerulopathy (C3G)	1	1.9

### Pathohistological analysis of all biopsied patients

Most of 54 biopsied cases underwent complete pathohistological analysis. The relative distribution of renal diseases diagnosed by biopsy is shown in Table 2.

The most common PHD-s were IgA nephropathy (IgAN) (24.1%), followed by minimal change disease (MCD) (16.7%). Henoch-Schonlein purpura nephritis (HSPN) was present in 14.8% of cases. Focal segmental glomerulosclerosis (FSGS) and Alport syndrome (AS) were each found in 11.1% of cases. Tubulointerstitial nephritis (TIN) and membranous glomerulopathy (MGN) were each 3.7%. The one patient that had FSGS also had TIN, while the one case with IgAN also had thin basement membrane nephropathy (TBMN). The one patient with MCD additionally had TBMN. Other cases were rarely diagnosed (Table 2).

In 8 (57.14%) out of 14 children with NS, biopsy was done because of steroid dependence, in 4 (28.57%) because of steroid resistance. One had infrequent relapses, while one had complete remission (Table 3). Out of 8 patients with SDNS, 7 (87.5%) had MCD, while one (12.5%) had FSGS. In the group of 4 patients with SRNS, three (75%) had FSGS and one (25%) had C1q nephropathy (C1qN).

**Table 3:** Distribution of response pattern to steroid therapy in patients in groups of *Pure nephrotic syndrome without hematuria* and *nephritic syndrome with nephrotic proteinuria* (*N* = 24)

Response to corticosteroids	Pure nephrotic syndrome without hematuria (N)	Nephritic syndrome and nephrotic proteinuria (N)	Total
Steroid dependant	8	3	11
Steroid resistant	4	0	4
Frequent relaps	0	0	0
Infrequent relaps	1	0	1
Complete remission	1	5	6
Not treated	0	1	1
Unknown	0	1	1
Total	14	10	24

When we combine data from patients with *NS* and *nephritic syndrome with nephrotic proteinuria* we found that out of 24 patients, there were 22 patients on steroid therapy. Among them, 11 had SDNS, 6 CRNS, 4 had SRNS, while 1 had IFRNS (Table 3).

The most common PHD-s included MCD (37.5%), HSPN (20.8%), and FSGS (16.7%). The detailed distributions are shown in Table 4.

**Table 4:** Frequency of different forms of pathohistological diagnosis in children with: *pure nephrotic syndrome without hematuria* and nephritic syndrome with nephrotic proteinuria (24 renal biopsies); pure nephrotic syndrome without hematuria, only (14 renal biopsies); nephritic syndrome with nephrotic proteinuria (10 renal biopsies)

Syndrome	Number	Percentage
Pure nephrotic syndrome without hematuria and nephritic syndrome with nephrotic proteinuria	**24**	
MCD	9	37.5
HSPN	5	20.8
FSGS	4	16.6
IgAN	2	8.3
MGN	2	8.3
C1qN	1	4.1
AS	1	4.1
Pure nephrotic syndrome without hematuria	**14**	
MCD	9	64.2
FSGS	4	28.5
C1qN	1	7.1
Nephritic syndrome with nephrotic proteinuria	**10**	
HSPN	5	50
MGN	2	20
IgAN	2	20
AS	1	10

*MCD* Minimal change disease, *FSGS* Focal segmental glomerulosclerosis, *C1qN* C1q nephropathy, *HSPN* Henoch-Schonlein purpura nephritis, *MGN* Membranous glomerulopathy, *IgAN* IgA nephropathy, *AS* Alport syndrome

### PHD-s in the group of NS

Among the 14 patients with *NS*, nine (64.28%) had MCD, four patients (28.57%) had FSGS, while C1q nephropathy was present in one (7.14%) case (Table 4).

Out of ten patients with *nephritic syndrome with nephrotic proteinuria*, five had HSPN, two (20.00%) had MGN, two (20.00%) patients had IgAN, while AS was present in one (10.00%) case (Table 4).

**Fig. 2:**
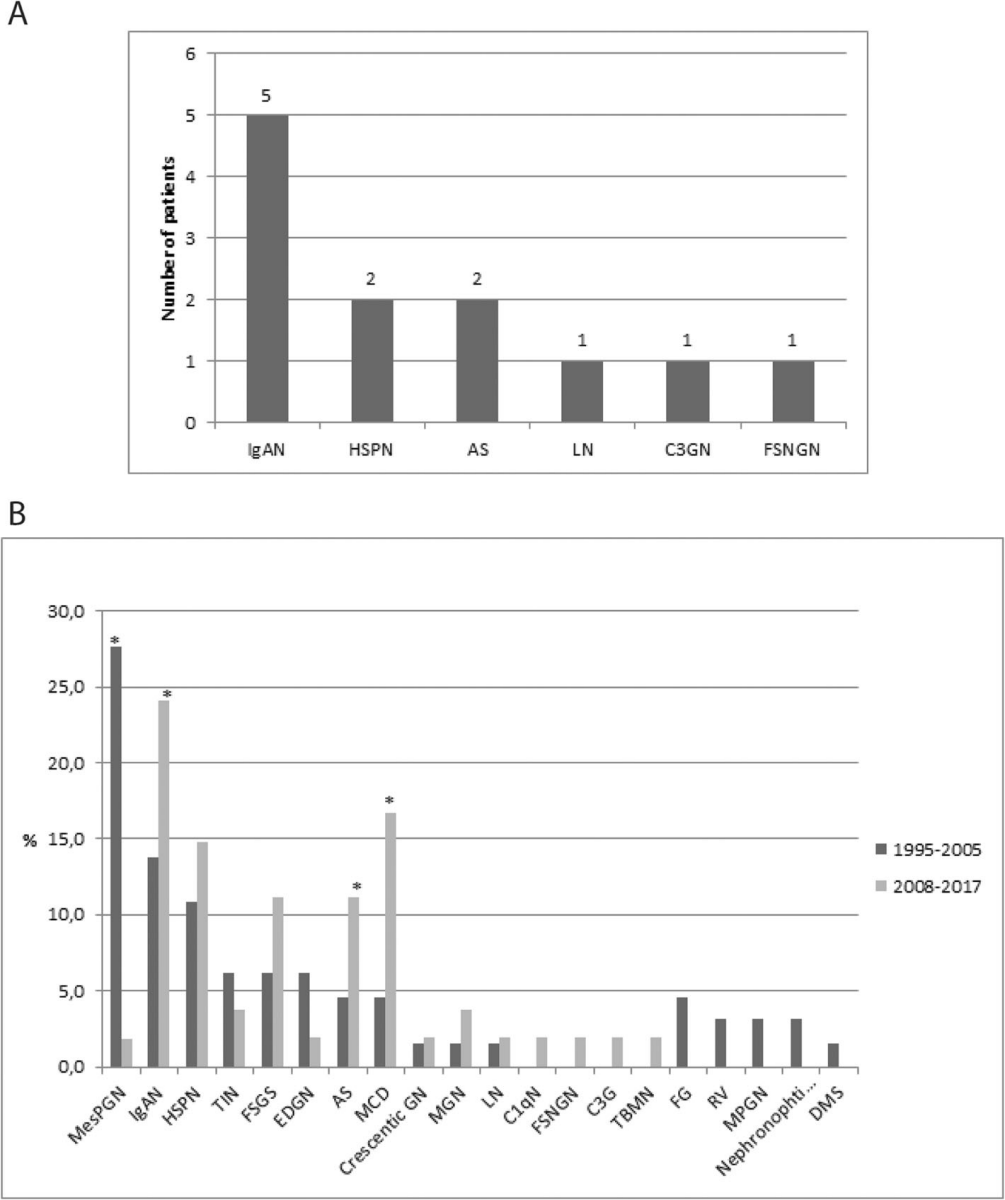
**a** Frequency of pathohistologic diagnoses in children with *non-nephrotic proteinuria with haematuria* (12 renal biopsies). **b** Changes seen in the epidemiology of renal disease in children between the periods 1995–2005 and 2008–2017. C3GN (C3 glomerulonephritis), Mesangioproliferative glomerulonephritis (MesPGN), IgA nephropathy (IgAN), Henoch-Schӧnlein purpura nephritis (HSPN), Tubulointerstitial nephritis (TIN), Focal segmental glomerulosclerosis (FSGS), Endoproliferative glomerulonephritis (EDGN), Alport syndrome (AS), Minimal change disease (MCD), Crescentic glomerulonephritis (Crescentic GN), Membranous glomerulopathy (MGN), Lupus nephritis (LN), C1q nephropathy (C1qN), Focal segmental necrotizing glomerulonephritis (FSNGN), C3 glomerulopathy (C3G), Thin basement membrane nephropathy (TBMN), Fibrillary glomerulonephritis (FG), Renal vasculitis (RV), Membranoproliferative glomerulonephritis (MPGN), Nephronophthisis, Diffuse mesangial sclerosis (DMS); *p < 0.05, 1995–2005 vs, 2008–2017

Out of 12 patients with *non nephrotic proteinuria with hematuria*, five patients (41.66%) had IgAN, two (16.66%) HSPN, two (16.66%) AS, one (8.33%) case of each, lupus nephritis (LN), C3 glomerulopathy (C3G) and focal segmental necrotizing glomerulonephritis (FSNGN) (Fig. 2a).

Out of 9 children with *persistent isolated hematuria*, IgAN was observed in 4 (44.44%) patients (one patient with IgAN additionally had TBMN), AS was diagnosed in three (33.33%), cases, while HSPN was found in one (11.11%) case, as well as one (11.11%) case of TBMN.

Four children were presented with *acute renal failure*. TIN was diagnosed in 2 of them, and other two diagnoses were crescentic GN and endoproliferative glomerulonephritis (EDGN).

Three patients with *chronic renal failure*, had the following PHDs: IgAN in one patient, FSGS in other patient while one patient had combination of FSGS and TIN.

In one patient with *non-nephrotic proteinuria*, mesangial proliferative glomerulonephritis (MesPGN) was found while in another patient with *acute nephritic syndrome*, IgAN was observed.

#### Complications of renal biopsy

Fourteen (26%) out of all 54 biopsies had clinically mild complications, mostly macrohematuria (8), microhematuria (2) and subcapsular haematoma (4), observed by ultrasonography, with no hemodynamic consequences, and no need for blood transfusions.

#### Comparison of kidney biopsy results between 1995 and 2005 and 2008–2017

When we compared two time periods 1995–2005 and 2008–2017, changes in epidemiology of children renal diseases displayed a significant increase of IgAN, MCD, and AS (*p* < 0.05). On the contrary, there were no significant differences noted in HSPN and FSGS frequencies. MesPGN showed a significantly decreasing pattern (*p* < 0.05), while EDGN showed a decreasing trend although not significantly (Fig. 2b). Between the years 1995–2005, we have reported diffuse mesangial sclerosis (DMS), fibrillary glomerulonephritis (FG), renal vasculitis (RV), membranoproliferative glomerulonephritis (MPGN) and nephronophthisis, diagnoses that we did not during the time period of 2008–2017. In contrast, in the last 10-year period, we reported C1q nephropathy, FSNGN, C3 glomerulopathy and TBMN, diagnoses that we did not have in the period between 1995 and 2005 (Fig. 2b).

**Fig. 3:**
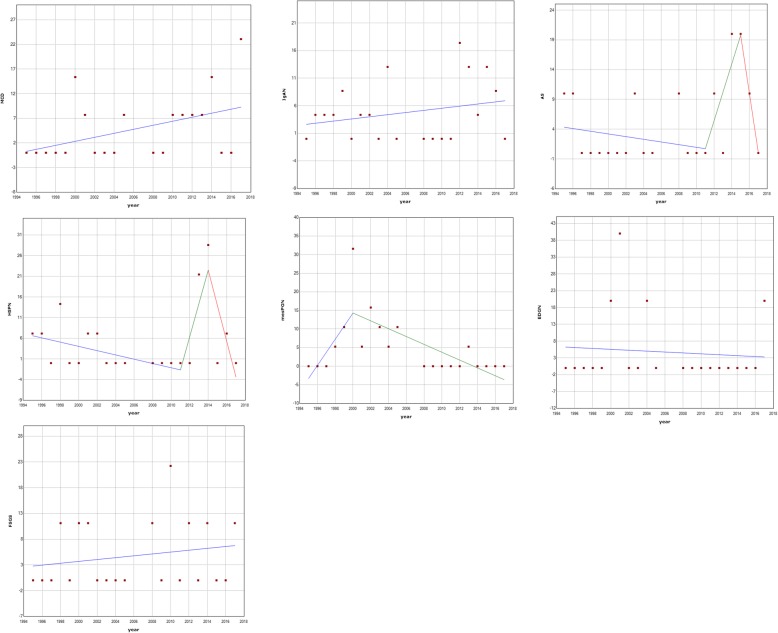
22-year long timeseries of relative frequencies with optimal jointpoint regression models for each pathologic entity. Mesangioproliferative glomerulonephritis (MesPGN), IgA nephropathy (IgAN), Henoch-Schӧnlein purpura nephritis (HSPN), Tubulointerstitial nephritis (TIN), Focal segmental glomerulosclerosis (FSGS), Endoproliferative glomerulonephritis (EDGN), Alport syndrome (AS), Minimal change disease (MCD)

When we analysed trends and breakpoints in whole time series of relative frequencies for each pathological entity from 1995 to 2017 few findings were noticed (Fig. 3). Increasing linear trend of 0.43% +/− 0.04% per year (*p* < 0.0001) was noticed for MCD. Similarly, IgAN showed a linear increase of 0.18% +/− 0.06% per year (*p* = 0.0059). Frequency of AS showed a sharp increase in 2011. (95%CI:1997. to 2012.) and fall in 2015. (95%CI: 2013. to 2015.). HSPN time series showed a similar pattern with a increase in 2011 (95%CI: 2005. to 2012.) and fall in 2014 (95%CI: 2012. to 2015.). MesPGN showed a steady increase of 3.51% +/− 1.08% per year (p = 0.005) until year 2000. (95%CI: 1998 to 2002), afterwards frequency of mesPGN is decreasing by 1.05% +/− 0.17% per year (*p* < 0.0001).

## Discussion

As in other similar studies, in our study there were slightly more boys than girls [5, 11–13, 28–32], while girls predominated only very rarely [16, 18]. The mean age at biopsy was about 10 years, which is corresponding to results reported in other countries [7–9, 11, 18, 22, 31]. However, sometimes that mean age was higher [15, 16] or lower [13, 28–30, 32].

Among 23 studies which have been analysed and compared to our study, only 9 used a similar technique and performed three different diagnostic techniques together: LM, IM and EM [1, 7, 9, 12, 17, 19, 22, 29, 33]. Eight studies did not used EM at all [11, 13, 16, 18, 20, 28, 30, 34], 5 used EM partly [2, 4, 6, 14, 15], while one used both IM and EM only partly [8]. The described technical heterogeneity could sometimes result in significant differences in PHD-s.

### Indications for renal biopsy

In accordance with the inclusion criteria, among the total number of patients with glomerular and tubulointerstitial diseases admitted to our Department during the investigated period, renal biopsy was performed in 23.4% of cases. Contrary to our results, the study by Fidan et al. reported kidney biopsy in about 10% of patients with renal diseases [3]. This difference might be attributed to still unclear/different inclusion criteria. The variations in indications for kidney biopsy can cause the diversities of PHD-s among many groups of patients all over of world. Therefore, comparison of PHD results sometimes might be difficult, even impossible. Moreover, in some cases it is very difficult to compare results of biopsies performed in the same centres during different periods of time, due to changes of indications for biopsy, methodology of tissue analysis, newly described diseases, and new diagnostic classifications of kidney diseases. The indications for renal biopsy seem to be quite different among different countries [2, 5, 7–9, 11, 13, 15, 16, 18, 20, 22, 28].

The most frequent reason for renal biopsy in our research, was *NS* (about one quarter). These results are consistent with some studies from literature [2, 7–11, 13, 15, 16, 18, 20, 22, 28], while in studies by Czech authors *isolated hematuria* was the main indication [4, 6]. *Nephrotic proteinuria with hematuria* was the main indications in Morocco [20], while in the study by authors from UK *non-nephrotic proteinuria* predominated with 36% of cases [10].

The reason for renal biopsy in *NS* group of patients regarding response to corticosteroid therapy gave predictable results due to the fact that we usually perform biopsy in patients failed to reach complete remission with corticosteroid therapy. The response to steroid therapy in SDNS patients was well at the beginning, but occasionally we needed to execute a renal biopsy due to corticosteroid side effects or because of the need to use another immunosuppressant. These findings are in correlation to studies done by Paripović et al., Kanodia et al., and Bazina et al. [9, 13, 16], while they differ from results of Printza et al. [18] and Boyer et al. [33]. In contrast, Bircan et al. had a very wide scale of indications for biopsy in patients with *NS*, with only 21.9% of SDNS, 13.2% of SRNS, and even 26.3% of patients with complete remission of *NS* [35]. In our study, the second indication for renal biopsy was *non-nephrotic proteinuria with hematuria*, which is in accordance with some other studies [1, 9, 22]. About one fifth of indications for renal biopsy in our research was *nephritic syndrome with nephrotic proteinuria,* which is significantly higher than in our previous studies, where that category was comprised of 3.1% of all biopsied cases or 10.8% in the study of Coppo et al. [2, 9].

Almost half of the indications for renal biopsy occur with combination of *NS* and *nephritic syndrome with nephrotic proteinuria*, which is mostly in line with previous studies [1, 9, 17]. In our study *isolated hematuria* was the fourth indication for renal biopsy with 16.7% of cases, which is slightly more than in our previous study (12.3%) [9]. Coppo et al. [2] and Rychlík et al. [6] found that *isolated hematuria* was the key indication for renal biopsy in children. This may be true because indications for renal biopsy in asymptomatic patients with microscopic hematuria are debatable. Study of Zhai et al. [36] suggests that isolated hematuria has relatively low risk of severe pathohistological lesions and that long-term monitoring is recommended, while Coppo et al. suggested that kidney biopsy should be made promptly [2].

In our study, *acute renal failure* was a less important reason for renal biopsy, while some studies reported similar [7, 9, 11, 37], higher (10.7–17.5%) [2, 10, 18, 20], or lower numbers (1.4–4.4%) [8, 15]. Similarly, *chronic renal failure*, was also less important reason for renal biopsy in our research.

By comparing different studies, we could show that indications for renal biopsy have changed throughout time. Thus, in the study of Yin et al., an increasing trend of NS and decreasing trend of acute glomerulonephritis (AGN), rapidly progressive glomerulonephritis (RPGN) and *isolated hematuria* had been described [22], while the study in Czech Republic displayed an increase of isolated haematuria and decrease of NS as a reason for renal biopsy [4]. Comparison of our two studies [2] showed almost the same percentages of NS and AGN, there was a modest increase of *isolated hematuria* and a decrease of *non- nephrotic proteinuria* as the indication for renal biopsy during that period.

The most common PHD diagnosis in our research was IgAN. These findings are similar to study done by Coppo et al. [2] and others [5, 6, 12, 21]. Contrary to our results, some studies found lower frequency of IgAN [1, 3, 8–10, 13–16, 18, 20, 34] or had not mentioned IgAN at all [28].

The reason for such differences could be the fact that IM was used in some studies only “when indicated” [8] or when the IgAN was expected [28]. MCD in our research, was the second most common PHD, while other authors found MCD as the first indication [5, 8, 11, 14, 20, 34]. The reason for this finding might by extensive indications for renal biopsy in NS in cimparison to our Hospital. FSGS was the most frequent PHD in Turkey, Greece and Serbia, probably due to stricter renal biopsy indications in NS patients in those countries [3, 16, 18]. In addition, biopsy practice in England showed that HSPN was the most common PHD (15.9%) [10], while MesPGN was the most frequent PHD in Southern Croatia in the period from 1995 to 2005 [9]. Those differences in PHD-s probably come from a different attitude towards patients with renal diseases, different indications for renal biopsy, possible different diagnostic methods of tissue analysis, and use of different pathohistological classifications.

MCD was the most common pathohistological finding in NS group of patients, and FSGS was the next. This is in agreement with other national studies [2, 4, 6, 7, 33]. The possible explanation for differences in PHD ranging could be the different policy towards renal biopsy in patients with NS. Therefore, in some countries renal biopsy indications are strict and renal biopsy is not an option when there is a response to corticosteroids.

### PHD-s regarding the response to corticosteroid therapy in the patients with *NS*

Our study showed that SDNS had MCD as PHD in about 90% of patients, and the remaining had FSGS. This might be attributed the hypothesis that if a patient responds to corticosteroids there is no indication for renal biopsy. In the group of our patients with SRNS, FSGS was the PHD in 75% of cases. High percentage of FSGS was also described by Kanodia et al. [13] and in the Greek study by Printza et al. [18]. Those results could be expected in view of the fact that these PHD-s are poorly treatable with corticosteroids.

In our research, IgAN was the most usual PHD, in the group of 12 patients with *non-nephrotic proteinuria with hematuria*. This finding was similar to findings reported in Japan [2, 38].

In the group of *nephritic syndrome with nephrotic proteinuria* from our study, 50% of PHD-s were HSPN, while Moorani et al. [28] and Zhou et al. found different distribution of PHD-s [32].

When we combined MCD, HSPN, and FSGS, these were the most frequent PHDs in our research. Contrarily, Zhou et al. found MesPGN, FSGS, IgAN as the most frequent PHDs [32]. However, those two studies are not comparable because in the study by Zhou et al. methodology for tissue analysis has not been mentioned at all.

### PHD-s in the group of patients with *isolated haematuria*

Among all cases of *isolated hematuria* from our study, IgAN was the most frequent pathohistologic diagnosis which is in accordance with results of Coppo et al. and Yin XL et al. [2, 22]. Some authors had IgAN in third place [36], but it is not clear whether they used IM in all analysed samples or not.

In half of the patients, TIN was usual PHD finding in *acute renal failure*. Crescentic GN was found only in one patient as well as EDGN. In contrast, a Spanish study by Lopez-Gomez et al. listed thrombotic microangiopathy as the main reason for renal biopsy, while in South Asian children HUS and TIN were also an important cause for AKI [15, 37]. A report from the Italian National Registry of Renal Biopsies in Children indicated that crescentic GN was the most common disease in the group of *acute renal failure*, followed by TIN [2].

Among our three patients with *chronic renal failure*, one had IgAN, one patient had FSGS as well as TIN, and one patient had FSGS, Mohapatra et al. also showed IgAN as a predominant diagnosis, followed by endoproliferative glomerulonephritis (EPGN) and FSGS [15]. The lack of data in the chronic renal failure group could be the reason for a small incidence in children. Additionally, kidney biopsy is not necessary to start appropriate treatment in advanced stage of certain chronic kidney disease.

### Trends of PHD results along the time

Our comparative study of PHD-s of all biopsies showed significant increase of IgAN from 13.8% in our previous study to 24.7% in this study [2]. Similar results were noticed in some other comparative studies as well [3, 12]. Those findings might be the result of higher numbers of biopsied patients with isolated hematuria and changes in classification of IgAN. With the use of Oxford classification of IgAN, many samples with previous PHD of MesPGN changed into IgAN, because mesangial proliferation is now a part of Oxford classification [23, 39, 40].

Czech Registries of Renal Biopsies from the period between 1994 and 2011, showed that MCD and minimal glomerular abnormalities were the most frequent PHD, and they have increasing pathways [4, 6]. In our two comparative studies, the frequency of MCD increased significantly from 4.6 to 16.7% [9]. Although these numbers show a significantly increasing trend, they are still relatively smaller than in Czech studies. We believe that this can be explained due to the fact that we had stricter indications for NS kidney biopsy in our hospital. The classification of proliferation of mesangial cells has also changed (previous study was done from 1995 to 2005) at which point MCD was named MesPGN. In our present study, the number of MesPGN dramatically dropped compared to our previous study [9]. High variability of MesPGN was also reported in other countries, such as in Pakistan [28], India [13], and Jordan [41]. The decreasing pathway of MesPGN in our research may be explained by some of the cases of IgAN that were unrecognized previously, due to incomplete sample analyses with immunohistochemistry and electron microscopy, or due to the introduction of updated Oxford classification of IgAN [4, 6, 23, 25, 31, 39, 40]. In that circumstance many IgAN diagnoses, as well as MCD and TBMN had a higher possibility to be missed or mixed up [6].

In our study the frequency of Alport’s syndrome significantly rose compared to our previous study [9]. The reason for that could be that we paid more attention to patients with isolated hematuria than earlier, due to growing knowledge on inherited glomerulopathies. Generally, we have to add that PHD of AS and TBMN can be confirmed by genetical analysis. Therefore, we expected that renal biopsy will not be necessary in many cases of hereditary glomerulopathies in the future. Biopsy will probably be done only in selected cases [42].

FSGS from our study shares the fourth and fifth place with AS, showing an increasing but not significant trend over time, when compared to our results from the previous 10-year period [9]. Similar results were reported in the study from China [22].

During the study period, we also noticed an insignificant increase of HSPN frequency, which was recorded in one Turkish and Chinese study, as well [3, 12]. Those findings are probably due to increased awareness of possible renal affection in Hennoch Schonlain vasculitis [31, 43]. Our investigation also showed a permanently low number of LN cases, as also shown in the research by Fidan et al. while LN is more common in Hispanics, Blacks and Asians than Caucasians [15, 20].

The prevalence of C1q nephropathy in renal biopsy worldwide has been shown to vary between 0.2 to 16% [44–47]. The reasons for this discrepancy could be attributed to inconsistent use of C1q immunofluorescence staining, and different criteria for the renal biopsies. In our study C1q was found in about 2% of all biopsied patients, which had not been noted in our previous study at al [9].

The drawback of our study is small numbers of renal biopsies to conclude a significant trend. Therefore, more cases should be observed in future studies to draw appropriate conclusions.

### Complications of renal biopsy

Regarding biopsy complications, their numbers vary significantly in literature from 3.0–30%, but very few publications reported serious complications like heavy bleeding or gross hematuria [7, 17]. Therefore, our study is in accordance with the other studies that had low frequency of severe complications [4, 6, 7, 9, 10, 17, 18].

### Comparation of biopsy results between two 10-years periods in our hospital

The significant rise in frequencies of IgAN, AS and MCD and insignificant rise of HSPN, FSGS in the recent 10-year period should be noticed, as well as significant decrease of MesPGN, compared to our previous study [9].

The reason for this difference might be the different PHD classification. Namely, for the classification of MesPGN we previously used the Churg criteria [48], which was common in the past, while new criteria is being used since 2006 [49]. Additionally, mesangial proliferation was associated with a loss of podocytes on EM, and was classified as MCDs with minimal proliferation of mesangial cells, or as IgM nephropathy in case of abundant IgM.

In our study, IgAN demonstrated a trend of increased frequency in comparison to the period of 1995–2005 primarily due to implementation of Oxford classification [23, 39, 40]. The reason for this might also be our tendency to select more patients with persistent microscopic hematuria, with or without associated proteinuria for renal biopsies. When clinical findings point to HSPN, biopsy was done to confirm the diagnosis and to predict the disease prognosis, which is important for therapy. Therefore, it was more often performed than previously.

## Conclusions

We found that the frequencies of MesPGN, IgAN, AS, MCD nephropathies in children in Croatia’s Dalmatia region has changed from the period 1995–2005 to 2008–2017. IgAN, AS, MCD nephropathies are more common and MesPGN nephropathy is diagnosed less frequently based on a kidney biopsy. Changes in the relative frequency of GN type might be partly due to the use of new classifications of certain glomerulonephritis (HSPN, IgAN) and the appearance of new entities (C3 nephropathy, C1q nephropathy). An additional reason for these changes may be due to policy changes and practices, changes in performing renal biopsies [9] as well as availability of technical possibilities for patohistological analysis of biopsied material, especially electron microscopy. All these factors can influence different prevalence of pathological findings. Therefore, it is necessary to continue monitoring renal biopsies in order to gain clinical knowledge important for establishing new guidelines that would help clinicians in everyday practice.

## Data Availability

The datasets used and/or analysed during the current study are available from the corresponding authors on reasonable request.

## Abbreviations

AGN: Acute glomerulonephritis
AKI: Acute kidney injury
AS: Alport syndrome
BIC: Bayesian information criterion
C1q: Complement component 1q
C1qN: C1q nephropathy
C3: Complement component 3
C3G: C3 glomerulopathy
C4: Complement component 4
CI: Confidence interval
crescentic GN: crescentic glomerulonephritis
CRNS: Complete remission nephrotic syndrome
DMS: Diffuse mesangial sclerosis
EM: Electron-microscopy
EPGN: Endoproliferative glomerulonephritis
FG: Fibrillary glomerulonephritis
FRNS: Frequently relapsing nephrotic syndrome
FSGS: Focal segmental glomerulosclerosis
FSNGN: Focal segmental necrotizing glomerulonephritis
GN: Glomerulonephritis
HSPN: Henoch-Schönlein purpura glomerulonephritis
HUS: Hemolytic–uremic syndrome
IFRNS: Infrequently relapsing nephrotic syndrome
IgA: Immunoglobulin A
IgAN: IgA nephropathy
IgG: Immunoglobulin G
IgM: Immunoglobulin M
IM: Immunofluorescence
LM: Light-microscopy
LN: Lupus nephritis
MCD: Minimal change disease
MesPGN: Mesangioproliferative glomerulonephritis
MGN: Membranous glomerulopathy
MPGN: Membranoproliferative glomerulonephritis
NS: Nephrotic syndrome
PHD: Pathohistological diagnoses
RPGN: Rapidly progressive glomerulonephritis
RV: Renal vasculitis
SDNS: Steroid dependent nephrotic syndrome
SRNS: Steroid resistant nephrotic syndrome
SSNS: Steroid sensitive nephrotic syndrome
TBMN: Thin basement membrane nephropathy
TIN: Tubulointerstitial nephritis
UHC Split: University Hospital of Split
WHO: World Health Organization
